# Corpulence

**Published:** 1884-04

**Authors:** 


### CORPULENCE.

WHATEVER we have written in reference to the means to be employed for reducing obesity, has always contained a caution against carrying our remedies to extremes. The golden rule of "moderation in all things," applies with especial force to this subject, since errors in the method of accomplishing the desired result may involve the sacrifice of the patient. A strict adherence to the rules published by "Banting," are certain to reduce corpulency, and at the same time to impair, if not destroy the health. It requires a wonderful amount of patience to remain contented while watching the slow processes of mild, but safe remedies for the cure of obesity; knowing that there is a shorter, even if more dangerous path that might be pursued. But if the sufferer from corpulency is not satisfied with slow results, he had better not attempt the treatment.

Deprived of all technical terms and obscure theories: a superabundance of fat is produced by eating more than is required for the legitimate wants of the system; and particularly of sugar, and starchy substances, as potatoes and wheat bread. It has been proved—contrary to the general belief on this subject—that eating fat in moderation does not produce fat. Prof. Ebstein, of Goettingen, Germany, has given this subject a thorough and scientific investigation; and he claims that the treatment of corpulency by regulating the diet, hardly involves any great self-denial on the part of the patient.

He allows a rather attractive bill of fare, with a variety of dishes, but positively and forever excludes potatoes and limits the quantity of bread. For breakfast he allows one cup of black tea without milk or sugar; about two ounces of white or brown bread and *plenty of butter*. Dinner—Soup (with bone marrow occasionally), five to six ounces of meat, boiled or stewed, with fat gravy; especially *fat* meat, plenty of vegetables, cabbage, and most of all, legumes (peas, beans). Beets, carrots and turnips are, on account of the sugar they contain, almost totally excluded—potatoes entirely. After dinner, a little fresh fruit, but without sugar. Supper—Tea without sugar or milk, one egg or a little fat meat, or both; or some ham with its fat, sausage, smoked or fresh fish, two ounces of white bread with plenty of butter, and occasionally a little cheese and a little fresh fruit.

A persistence in this plan for a few months, we are assured, will reduce corpulence; but there must be no going back to old habits, or the trouble will return. This bill of fare or a similar one must be maintained during life.

